# Xanthohumol and its non-estrogenic derivatives link to the gut-liver-brain axis to improve cognition in mice with diet-induced obesity

**DOI:** 10.3389/fphys.2026.1886058

**Published:** 2026-08-05

**Authors:** Alexandra Alexiev, Keaton Stagaman, Kristin Kasschau, Yang Zhang, Jacob Raber, Adrian F. Gombart, Claudia S. Maier, Jan F. Stevens, Thomas J. Sharpton

**Affiliations:** 1 Department of Microbiology, Oregon State University, Corvallis, OR, United States; 2 Linus Pauling Institute, Oregon State University, Corvallis, OR, United States; 3 School of Biological and Population Health Sciences, Nutrition Program, Linus Pauling Institute, Oregon State University, Corvallis, OR, United States; 4 School of Medicine, Oregon Health and Science University, Portland, OR, United States; 5 Department of Biochemistry and Biophysics, Oregon State University, Corvallis, OR, United States; 6 Department of Chemistry, Oregon State University, Corvallis, OR, United States; 7 Department of Pharmaceutical Sciences, Oregon State University, Corvallis, OR, United States; 8 Department of Statistics, Oregon State University, Corvallis, OR, United States

**Keywords:** bile acid (BA) metabolism, ceramide metabolism, cognition, gut metagenome, high-fat diet, tetrahydroxanthohumol, xanthohumol (PubChemCID: 639665), α,β-dihydro-xanthohumol

## Abstract

Obesity-associated cognitive decline represents a growing public health concern, yet the mechanisms linking high-fat diet (HFD) to neurological impairment remain incompletely understood. Xanthohumol (XN) and its non-estrogenic derivatives, tetrahydroxanthohumol (TXN) and α,β-dihydro-xanthohumol (DXN), improve metabolic dysfunction and cognitive impairment associated with diet-induced obesity. The mechanisms underlying these cognitive benefits remain poorly defined, but all three compounds improve glucose tolerance, spatial learning and memory in obese C57BL/6J mice. We hypothesized that the gut-liver-brain axis associates with these effects through modulation of gut microbial functional capacity and host ceramide metabolism. To test this, we integrated shotgun metagenomes with lipidomic and behavioral data from male C57BL/6J mice fed a HFD supplemented with XN, TXN, or DXN to determine (1) whether supplementation differentially alters gut metagenome functional capacity, (2) whether variation in the gut metagenome links to cognitive outcomes, and (3) whether supplementation-induced variation in the gut metagenome is associated with alterations in ceramide and bile acid levels in the liver and hippocampus. We found that microbial gene abundance was associated with spatial learning outcomes across all treatment groups, including genes involved in tryptophan metabolism. Gut microbiome composition was also linked to ceramide levels in both hepatic and hippocampal tissues, with C22 ceramide emerging as a shared biomarker. TXN supplementation additionally reduced secondary bile acids HDCA and a DCA-isomer, extending prior 16S rRNA-based findings to the level of microbial gene function. Collectively, these results are consistent with a model in which XN and its derivatives act upon the gut-liver-brain axis to improve cognition in obese mice in association with changes to gut microbial functional capacity (most notably in bile acid and ceramide metabolism, with tryptophan metabolism as a secondary observation).

## Introduction

Xanthohumol (XN) is a compound derived from hops that has recently been investigated as a supplement for improving health outcomes related to several high fat diet (HFD)-induced disorders (Bradley et al., 2020; Kundu et al., 2022; Miranda et al., 2016; Paraiso et al., 2021; Zhang et al., 2021). Such disorders include hepatosteatosis (Paraiso et al., 2021; Zhang et al., 2021), impaired glucose and lipid metabolism related to obesity (Miranda et al., 2016; Rossi et al., 2019; Yui et al., 2014), diabetes (Li et al., 2023; Miranda et al., 2018; Rossi et al., 2019), and metabolic syndrome (MetS) (Zhang et al., 2020). However, bio-transformation of XN into various metabolites could contribute to liver injury and/or exert estrogenic effects (Miranda et al., 2018; Possemiers et al., 2006). Previously, we showed that non-estrogenic XN derivatives tetrahydro-XN (TXN) and α,β-dihydro-XN (DXN) improve outcomes in obese mice without these associated risks (Miranda et al., 2018; Paraiso et al., 2021, 2020; Zhang et al., 2020).

Additionally, supplementation of HFD-fed mice with XN, DXN and TXN mitigates obesity-associated cognitive decline in our past studies (Kundu et al., 2022; Miranda et al., 2018; Paraiso et al., 2020). Independent studies likewise report cognitive benefits of XN, including improved Morris water maze performance and reduced neuroinflammation in rodent models of cognitive impairment (Długosz et al., 2025; Zamzow et al., 2014). These improvements correlated with decreased ceramides in the brain and liver, changes in bile acid synthesis, and improved liver function (Paraiso et al., 2021). Ceramides are bioactive lipids whose elevated levels have been consistently linked to high-fat diet, obesity, insulin resistance, and cognitive impairment (Bikman and Summers, 2011; Kundu et al., 2022; Paraiso et al., 2020; Summers, 2006; Xie et al., 2017; Zimmermann et al., 2001). They play a critical role in the development and functioning of the central nervous system and in the regulation of bile acid synthesis, fat storage, and inflammation pathways (Quinville et al., 2021). Thus, ceramide metabolism may serve as a mechanistic link between metabolic and cognitive dysfunction through interconnected pathways in the brain and liver (Bikman and Summers, 2011; Quinville et al., 2021).

Our prior work demonstrated that XN and its derivatives modulate the gut microbiome and secondary bile acid metabolism by effects on specific microbial taxa (Zhang et al., 2020). More broadly, the microbiome generates diverse classes of signaling metabolites, including tryptophan derivatives, that engage host pathways relevant to XN’s physiological effects (Appleton, 2018; Dehghani et al., 2025; Karim et al., 2023; Lindell et al., 2022; Newman et al., 2023; Vaher et al., 2022; Weersma et al., 2020; Wellington et al., 2022; Zhang et al., 2020). Specific taxa are altered by the consumption of XN (Bradley et al., 2020; Cermak et al., 2017; Lee et al., 2024) and its derivatives (Zhang et al., 2020), and XN’s physiological effects depend on gut microbiome composition (Logan et al., 2021; Newman et al., 2023; Zhang et al., 2020). Building on our group’s research implicating the gut-liver-brain axis in XN metabolism (Kundu et al., 2022, 2022; Miranda et al., 2018; Paraiso et al., 2021, 2020; Zhang et al., 2020), we hypothesize that the gut microbiome contributes to the cognitive changes observed in HFD and XN supplementation studies. The gut-liver-brain axis has been previously implicated in mood disorders and cognitive function (Appleton, 2018; Vaher et al., 2022), and gut microbiota affect drug and supplement metabolism by the liver (Dehghani et al., 2025; Karim et al., 2023; Lindell et al., 2022; Weersma et al., 2020; Wellington et al., 2022). Beyond composition, gut microbial *functional* capacity shows reproducible shifts in obesity across large, independent metagenomic cohorts (Chanda and De, 2024), and diet-induced obesity has been linked to cognitive decline through gut dysbiosis in independent rodent models (Mabrok et al., 2024). However, we have only recently connected the gut microbiome to XN-mediated health benefits, and prior 16S-based analysis of microbial community composition suggests a gene-specific approach could reveal underlying mechanisms.

We propose that XN metabolites may influence cognition by altering the functional capacity of the gut microbiome, thereby modulating liver and central nervous system function through changes in ceramide metabolism. To test this hypothesis, we leveraged metagenomic tools to identify microbial gene pathways associated with XN metabolites and to determine how these pathways link with cognitive outcomes. We further integrated these data with host liver and hippocampal lipidomic data, focusing on ceramides already linked to XN metabolism and cognition (Paraiso et al., 2020). This strategy was designed to identify candidate molecular pathways through which XN and its metabolites, gut microbiota, and host physiology interact to shape cognitive endpoints. Together, these analyses clarify the potential role of the gut microbiome in linking the cognitive benefits of XN and its derivatives and inform future mechanistic studies.

## Materials and methods

### Sample collection and experimental design

We collected data for sphingolipid concentrations, fecal bile acids levels, and spatial learning outcomes from our prior studies, which compared the effects of XN and its derivatives on insulin resistance, spatial learning and memory, and bile acid metabolism (Miranda et al., 2018; Paraiso et al., 2020; Zhang et al., 2020). Full details of the original animal study are described in Miranda et al. (2018); we summarize the design here. Nine-week-old male C57BL/6J mice (The Jackson Laboratory, Bar Harbor, ME, USA) were housed individually in plastic cages on a 12:12-h light–dark cycle and randomly assigned to four groups of 12. Control mice were fed a high-fat diet (Dyets Inc., Bethlehem, PA, USA; 60%, 20%, and 20% of calories from fat, carbohydrate, and protein), and the remaining groups received the same HFD containing XN, DXN, or TXN. Test compounds were dissolved in an oleic acid:propylene glycol:Tween 80 vehicle (0.9:1:1 by weight; OPT) before incorporation into the diet, and the control diet contained an equivalent amount of OPT; all diets were prepared in pellet form (Dyets Inc.) to deliver 30 mg compound/kg body weight/day. Food intake was recorded three times weekly and body weight weekly. After 13 weeks of feeding, mice were euthanized and tissues collected for downstream analyses. All procedures were approved by the Oregon State University Institutional Animal Care and Use Committee (IACUC-2019-0001). Sample sizes were determined by the prior *in vivo* studies from which these samples were derived **Table 1**).

**Table 1:** Total number of mice analyzed for each data type (gut metagenomics, fecal bile acids, spatial learning, and liver and brain sphingolipids), per treatment group (HFD, HFD + XN, HFD + DXN, HFD + TXN).

Treatment group	Number of mice for gut metagenomic sampling	Number of mice for fecal bile acids	Number of mice for spatial learning tests	Number of mice for liver sphingolipids	Number of mice for brain sphingolipids
Data source	Newly generated	(Zhang et al., 2020)	(Miranda et al., 2018)	(Paraiso et al., 2020)	(Paraiso et al., 2020)
HFD	12	12	12	11	11
HFD + XN	12	12	12	11	11
HFD + DXN	12	12	12	12	12
HFD + TXN	11	11	11	10	9
**Totals**	**47**	**47**	**47**	**46**	**43**

Citations for previously generated data are shown in data source.Total values are bolded for emphasis to make it easier to find them in the table.

We newly generated metagenomic data from fecal samples collected from mice and flash frozen in liquid nitrogen and stored at -80°C at the conclusion of the feeding study. We extracted genomic DNA from 2–3 fecal pellets and prepared it for sequencing as described previously (Zhang et al., 2020). Shotgun metagenomic sequencing (2x150 bp paired end reads) was performed by the Center for Quantitative Life Sciences at Oregon State University on an Illumina HiSeq 3000 sequencer (San Diego, California, USA).

Spatial learning data were derived from our prior published study (Miranda et al., 2018), which we describe here briefly. Time to reach the platform (latency) was assessed by training mice to locate a visible or hidden platform in a water maze. Mice were first trained to locate a visible platform in four trials, in which an escape platform was marked with a visual cue (L_Vis1, L_Vis2, L_Vis3, L_Vis4). During the visible platform trials, average swim speeds were also measured (SS_Vis). Spatial learning was assessed during seven hidden platform trials, in which mice must remember and locate the position of a submerged escape platform using extra maze cues (L_Hid1, L_Hid2, L_Hid3, L_Hid4, L_Hid5, L_Hid6, L_Hid7). To test spatial memory retention, the day following the last hidden platform trial, mice were administered a probe trial (no platform). Investigators were blinded to treatment group during behavioral testing and scoring; group assignments were unblinded only after all data had been analyzed. For analysis, the maze was divided in four quadrants and the time spent in the quadrant that contained the platform during the hidden platform trials (P_TargetQ), latency to first reach the platform location in the probe trial (P_Time), and the number of times the mice crossed the platform location were analyzed (P_Cross). Each metric was evaluated for 47 total animals **Table 1**).

We analyzed several sphingolipid and secondary bile acid concentrations that we hypothesized could be tied to XN metabolism and cognition (Kundu et al., 2022; Paraiso et al., 2020). Lipid and bile acid extraction and measurement of these samples is described in previous publications (Kundu et al., 2022; Paraiso et al., 2021, 2020; Zhang et al., 2020). Sphingolipid data were acquired using LC-MS/MS on a hybrid triple quadrupole linear ion trap mass spectrometer (AB SCIEX, 4000 QTRAP) (Paraiso et al., 2020). Bile acids were identified using Waters ACQUITY UPLC I class system coupled to a Waters Synapt G2 HDMS mass spectrometer (Zhang et al., 2020). We analyzed C16 ceramide, C18 ceramide, C20 ceramide, C22:1 ceramide, C22 ceramide, C24 ceramide, C24:1 ceramide, C18 SM, C20 SM, C22 SM, C24 SM, C18:1 SM, C22:2 SM, C22:1 SM, and C24:1 SM from hippocampal samples (Paraiso et al., 2020). We also analyzed sphinganine d16:0, sphingosine d18:1, sphingosine d20:1, C16 ceramide, C18 ceramide, C20 ceramide, C22 ceramide, C24 ceramide, C16 sphingomyelin, C18 SM, C20 SM, C22 SM, C24 SM, C16:1 SM, C18:1 SM, C20:1 SM, C22:1 SM, and C24:1 SM (Paraiso et al., 2020). Eleven bile acids were analyzed from feces and liver samples (Zhang et al., 2020).

### Shotgun metagenome data pre-processing

We used the BioBakery workflow (Beghini et al., 2021) to process shotgun metagenomic reads into a gene abundance table as follows. We used kneaddata to remove contaminant reads that match the host, *M. musculus*, (via Bowtie2) (Langmead and Salzberg, 2012) and trim sequences (via Trimmomatic) (Bolger et al., 2014). Taxonomy was assigned using Kraken2 (Wood et al., 2019). We aligned sequences to reference sequences (KEGG Orthologs; KOs) in the Kyoto Encyclopedia of Gene and Genomes (KEGG) database (release 94) (Kanehisa, 2019; Kanehisa et al., 2025; Kanehisa and Goto, 2000). KOs represent a gene sequenced from the microbiome sample with a known function. Together, these genes embody the functional capacity of the gut microbiome; they may or may not undergo transcription but the capacity for that gene function exists within the microbial community. The sequences can be found on NCBI’s database at BioProject PRJNA1477656.

### Statistical approach

We used R (version 4.0.0) to test three models evaluating the beta diversity of KOs in relation to our hypotheses: (1) treatment alone, (2) the interaction between treatment and spatial learning, and (3) the interaction between treatment and ceramides. All code can be found at https://github.com/aalexiev/XanthohumolMicrobiome.

We evaluated Bray-Curtis, Canberra, and Sørensen dissimilarities, and report the outcomes from the significant metrics per analysis in main text. A PCoA was used to visualize the effect of treatment on beta diversity of KOs, and PERMANOVA was used to determine significance. For the other two (interactive) models, dbRDA was used to visualize a model that was optimized using capscale(). We determined significance by using this optimized model formula as the input formula for PERMANOVA.

**Figure 1:**
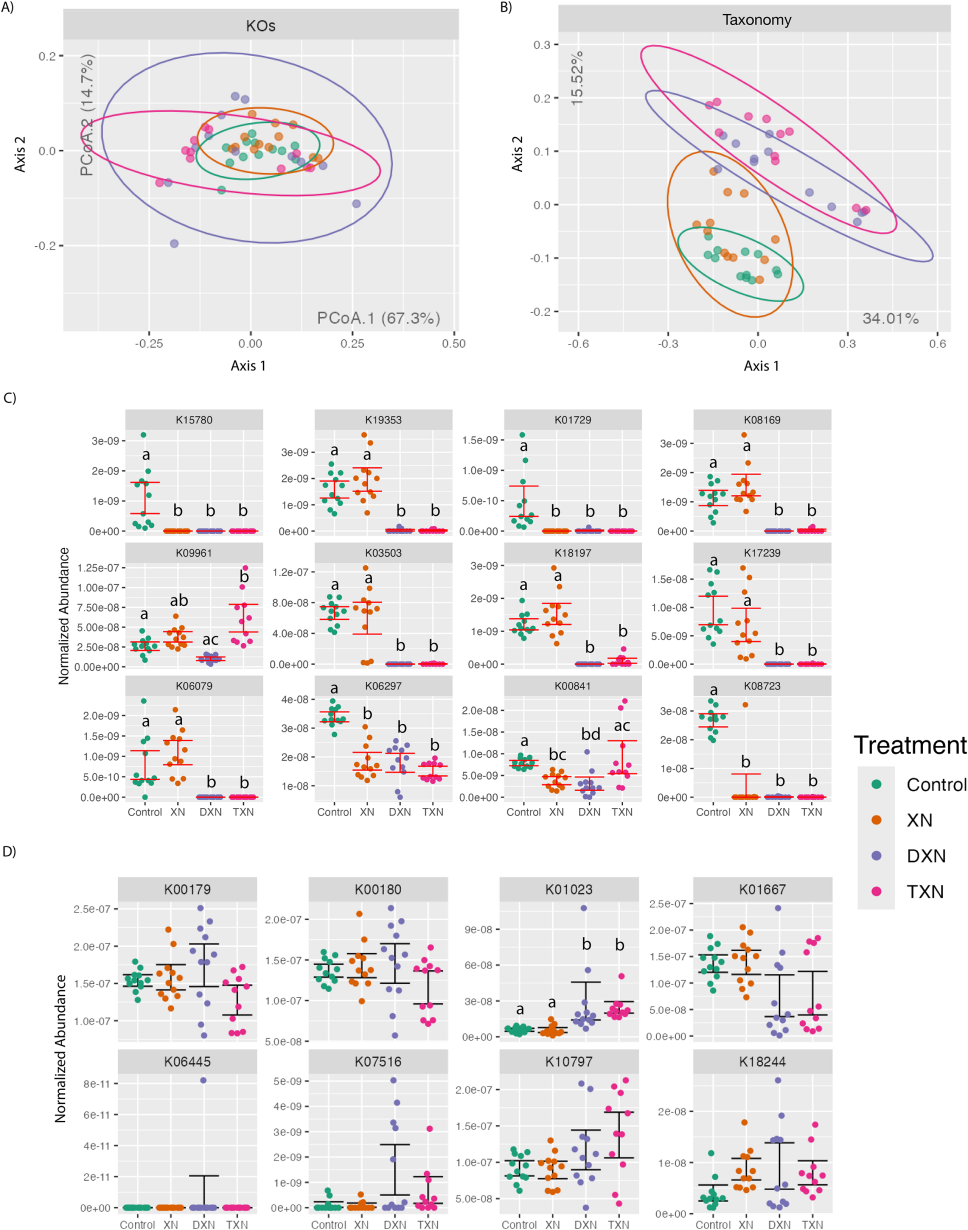
XN, DXN, or TXN treatment affects KOs and taxonomy of mouse gut metagenome. **(A)** PCoA ordination of Bray-Curtis dissimilarity between mouse metagenome samples with KO functional annotations. Ellipses encompass 95% of the data points in each diet treatment. Color represents treatment. **(B)** PCoA ordination of Canberra dissimilarity between mouse metagenome samples with taxonomic composition assigned. Ellipses encompass 95% of the data points in each diet treatment. Color represents dietary treatment. **(C)** Top 12 most important KOs for predicting mouse diet according to random forest models that were also significant according to Kruskal-Wallis tests (Bonferroni corrected, p ≤ 0.05). Significance is shown with letters indicating compound groups for significance. **(D)** Abundance by diet treatment for KOs whose description matches key terms identified as associating with XN (or derivatives) in prior literature. Significant comparisons are shown with letters indicating compound groups for significance (Kruskal-Wallis, Bonferroni corrected, p ≤ 0.05).

**Figure 2:**
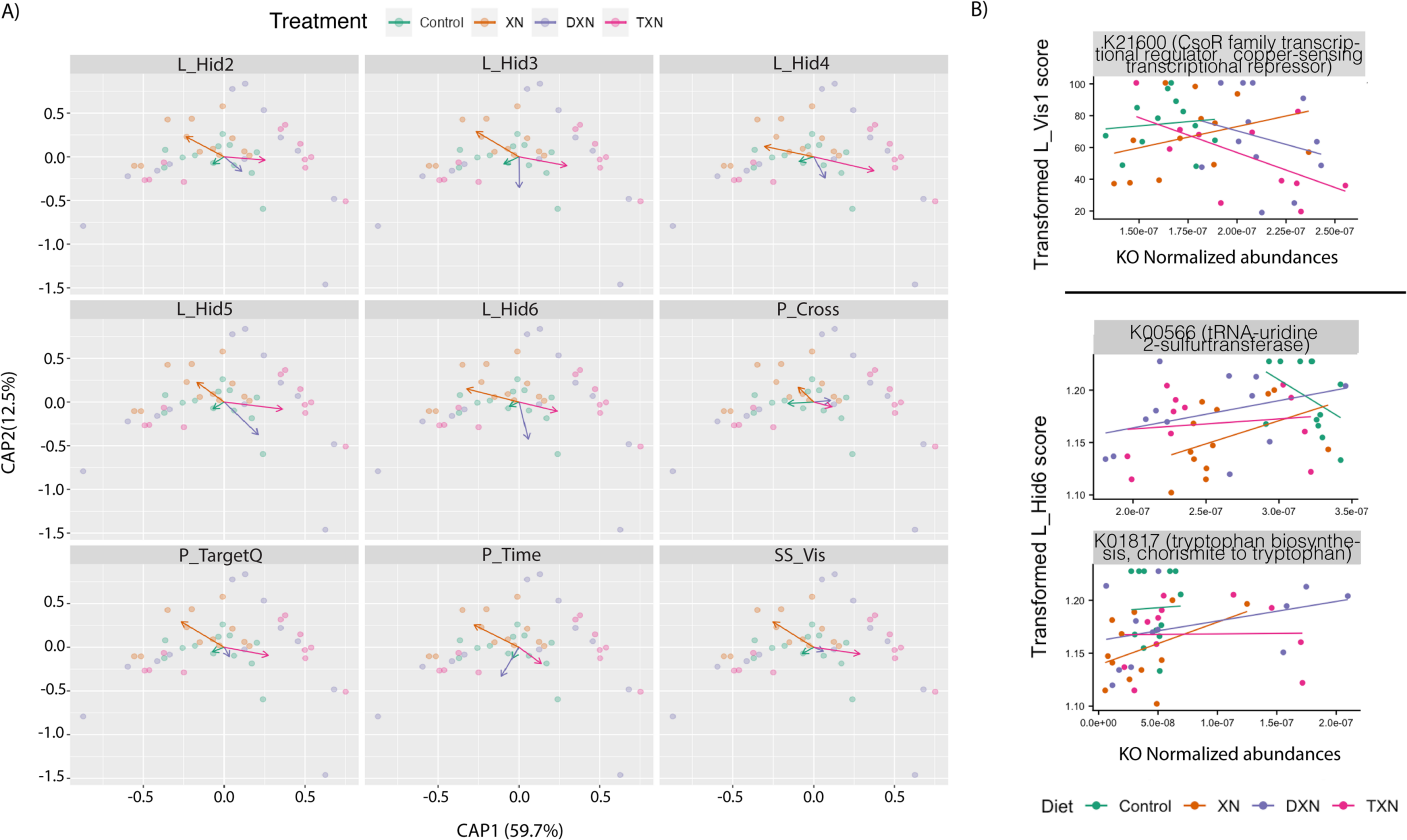
Gut microbiome gene function correlates with spatial learning, a measure of cognition, in a treatment-dependent manner. **(A)** dbRDA ordinations of Bray-Curtis distances of KO composition. Arrows indicate vectors of greatest change for the significant spatial learning covariate (indicated by the panel label) by treatment interaction term from the PERMANOVA results (significance of p ≤ 0.05). Both points and arrows are colored by treatment. Percent variance in Bray-Curtis distance explained is presented in parentheses in the axis labels. **(B)** Plots of standout example KOs whose abundances varied with treatment interacting with behavior metrics. KO normalized abundance is on the x-axis, and the related spatial learning scores that were dependent on treatment (color) are on the y-axis. Lines are shown that represent the best fit line for each relationship between KO abundance and behavior scores, per diet.

**Figure 3:**
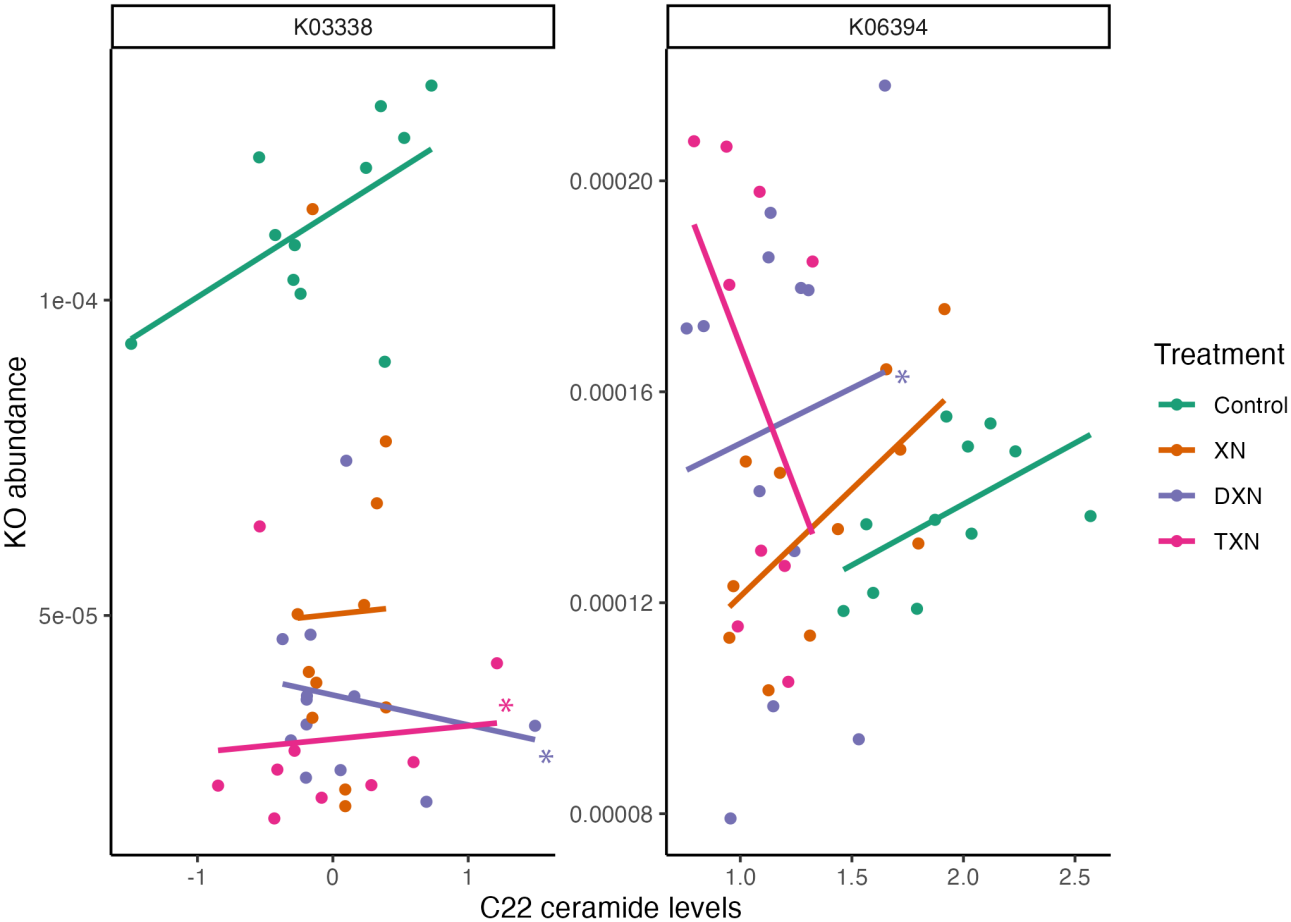
KOs significantly predicting C22 ceramide levels in the liver and brain. C22 ceramide was selected because it was the only ceramide associated with microbiome beta diversity in both brain and liver (see Results). KOs were identified first by a random forest regression model, then verified with a Gaussian linear model using the formula KO abundance ~ C22 ceramide levels + treatment + C22 ceramide levels:treatment. Asterisks represent significant linear regression p-values compared to control, and colors represent treatment type.

Sample sizes were fixed by the prior *in vivo* studies **Table 1**) and were not powered for metagenome-wide discovery. To mitigate this constraint, we used random forest as a feature-reduction step and independently validated selected genes with conventional significance testing (Kruskal-Wallis or Gaussian regression), and we interpret the resulting associations as hypothesis-generating rather than definitive. We trained random forest models on the full dataset using 5-fold cross-validation (caret package), tuning hyperparameters (mtry, splitrule) by AUC. Feature selection stability was done via the Altmann method (Altmann et al., 2010). The best random forest classification model identifying KOs describing treatment had a 15% out-of-bag error, 1.00 AUC of the ROC, 97% mean sensitivity, 99% mean specificity, 97% mean precision, and 98% mean accuracy. Because random forest served as a feature-reduction step rather than a predictive model, the high training AUC reflects in-sample separation among selected features and should not be interpreted as an estimate of out-of-sample predictive performance. We trained a random forest regression model predicting spatial learning; **Supplementary Table 1** shows the RMSE and other characteristics used to find the best fit for each behavior metrics. Lastly, we also ran random forest regression models to determine which KOs were associated with treatment and ceramide levels. Model RMSE and other characteristics can be found in **Supplementary Table 1**. We then ran subsequent models on these putatively predictive KOs to calculate significance (alpha = 0.05) and effect size for KOs describing treatment (Kruskal-Wallis), spatial learning (Gaussian linear regression), or ceramide levels (Gaussian linear regression) as relevant to **Figures 1C**, **2B**, and **3**.

**Figure 4:**
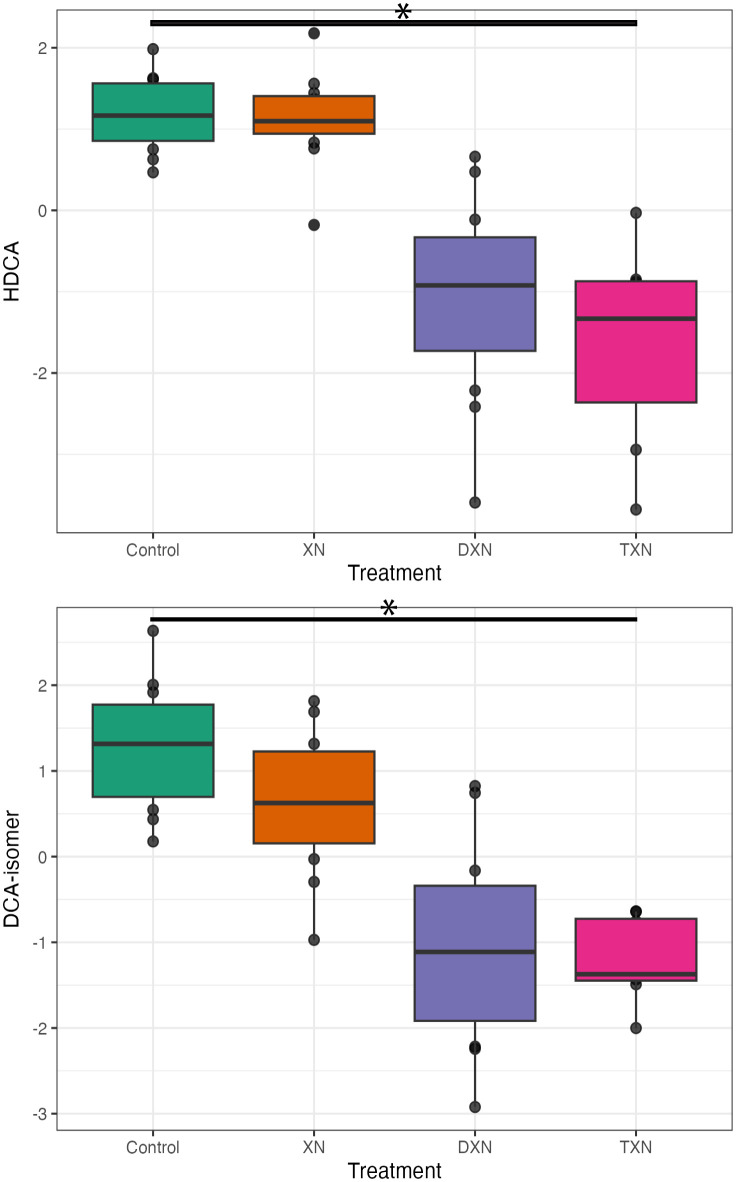
Boxplots of secondary bile acids that associated significantly with treatment. The y-axis shows the normalized abundances of two microbially-produced bile acids, HDCA and DCA-isomer. The x-axis and corresponding colors represent the treatments. Only TXN shows a significant shift from control, which is shown with an asterisk (Gaussian linear model, bile acid abundance ~ treatment * behavior). Significance was assessed using Benjamini-Hochberg FDR-corrected p-values; asterisks denote treatment groups differing significantly from control (FDR < 0.05).

Multiple-testing correction was applied per analysis: Kruskal-Wallis tests of diet-associated KOs (**Figures 1C, D**) were Bonferroni-corrected, and the bile acid models (bile acid ~ treatment * behavior; **Figure 4**) were corrected using the Benjamini-Hochberg false discovery rate across all model terms and bile acid-behavior combinations tested. PERMANOVA analyses (**Tables 2**; **3**) and the random-forest-selected KO-covariate linear models (**Figures 2B**, **3**) are reported at an uncorrected α = 0.05 on random-forest-selected features and interpreted as hypothesis-generating.

**Table 2:** Significant PERMANOVA results for Bray-Curtis dissimilarity by treatment and spatial learning covariate interactions for KO gene annotations from mouse gut metagenomes.

Term	df	Sum of squares	Statistic	P value
Treatment: SS_Vis	4	0.10	2.95	0.021
Treatment: L_Hid2	4	0.13	3.68	0.008
Treatment: L_Hid3	4	0.23	6.68	0.001
Treatment: L_Hid4	4	0.16	4.72	0.003
Treatment: L_Hid5	4	0.10	2.79	0.028
Treatment: L_Hid6	4	0.20	5.71	0.001
Treatment: P_Time	4	0.13	3.75	0.007
Treatment: P_Cross	4	0.17	5.10	0.001
Treatment: P_TargetQ	4	0.13	3.71	0.005
Residual	7	0.06		

Of note, visible trials were tested but did not yield significant associations.

**Table 3:** PERMANOVA results for Bray-Curtis dissimilarity of KOs in the gut by interaction between treatment and concentrations of brain and liver ceramides.

Term	df	Sum of squares	Statistic	P value
Treatment:brain_C20	4	0.17	4.42	0.001
Treatment:brain_C22:1	4	0.16	4.01	0.001
Treatment:brain_C22	4	0.22	5.65	0.001
Treatment:brain_C24	4	0.16	4.16	0.001
Treatment:liver_C16	4	0.14	3.68	0.003
Treatment:liver_C22	4	0.17	4.33	0.001
Residual	11	0.11		

## Results

### Supplementation with XN compounds affects the mouse gut metagenome

We first evaluated whether microbiome-derived genes (i.e., KOs) and taxonomic beta diversity was associated with treatment. Treatment explained Bray-Curtis dissimilarity of KOs in mouse gut (PERMANOVA: R2 = 0.42, p = 0.001), with DXN and TXN being the most dissimilar from the control (**Figure 1A**). Taxonomic diversity (particularly as measured by the Canberra distance) showed a similar ordination pattern to the KO data: diet treatments explained dissimilarity between gut taxonomic composition in mice, with DXN and TXN being the most dissimilar from the control (PERMANOVA, R2 = 0.25, p = 0.001) (**Figure 1B**). The Canberra distance is an abundance weighted metric like Bray-Curtis dissimilarity, but its metric is less influenced by highly abundant species. The fact that Sørensen (a presence-absence metric) and Canberra are much more significant in partitioning the metagenomic composition by diet treatment (**Supplementary Table 2**).

We next sought to determine which KOs were associated with treatment. A random forest model found that several KOs were associated with treatment (**Figure 1C**; **Table 4**). These fell into two categories that were relevant to our hypotheses. The first category were KOs whose abundances differed between control and supplemented (XN, DXN, and TXN) diets, representing potential functions that were altered as a result of treatment with any XN compound. Examples include K15780 (bifunctional protein TilS/HprT) or K06297 (spore germination protein). The second category were KOs which were abundant in the control and XN mouse metagenomes but not those of the XN derivatives, and likely drove the patterns seen in the beta diversity ordinations, such as K06079 (copper homeostasis lipoprotein). We also specifically tested the KOs in the following categories that we hypothesized could associate with XN or its derivatives: peptide fermentation (indolepyruvate ferredoxin oxidoreductase), phenol metabolism (arylsulfate sulfotransferase), tryptophan metabolism (tryptophanase), fatty acid oxidation (acyl-CoA dehydrogenase, 3-hydroxyacyl-CoA dehydrogenase, acyl-CoA dehydrogenase, 2-enoate reductase) (**Figure 1D**; **Table 4**). Among these, one was statistically significant: K01023 (arylsulfate sulfotransferase) is higher in DXN and TXN compared to XN and control diets (**Figure 1D**; **Table 4**).

**Table 4:** Important KOs that vary between different supplementation regimes, based on **Figures 1C, D**.

KO number	KO description	Corresponding figure panel and statistical significance (asterisk)
K00841	patA; aminotransferase [EC:2.6.1.-]	Figure 1C*
K01729	algL; poly(beta-D-mannuronate) lyase [EC:4.2.2.3]	Figure 1C*
K03503	umuD; DNA polymerase V [EC:3.4.21.-]	Figure 1C*
K06079	cutF, nlpE; copper homeostasis protein (lipoprotein)	Figure 1C*
K06297	gerKC; spore germination protein KC	Figure 1C*
K08169	yebQ; MFS transporter, DHA2 family, multidrug resistance protein	Figure 1C*
K08723	yjjG; pyrimidine 5’-nucleotidase [EC:3.1.3.-]	Figure 1C*
K09961	K09961; uncharacterized protein	Figure 1C*
K15780	tilS-hprT; bifunctional protein TilS/HprT [EC:6.3.4.19 2.4.2.8]	Figure 1C*
K17239	inoG; inositol-phosphate transport system permease protein	Figure 1C*
K18197	yesW; rhamnogalacturonan endolyase [EC:4.2.2.23]	Figure 1C*
K19353	eptC; heptose-I-phosphate ethanolaminephosphotransferase [EC:2.7.8.-]	Figure 1C*
K00179	iorA; indolepyruvate ferredoxin oxidoreductase, alpha subunit [EC:1.2.7.8]	**Figure 1D**
K00180	iorB; indolepyruvate ferredoxin oxidoreductase, beta subunit [EC:1.2.7.8]	**Figure 1D**
K01023	assT; arylsulfate sulfotransferase [EC:2.8.2.22]	Figure 1D*
K01667	tnaA; tryptophanase [EC:4.1.99.1]	**Figure 1D**
K06445	fadE; acyl-CoA dehydrogenase [EC:1.3.99.-]	**Figure 1D**
K07516	fadN; 3-hydroxyacyl-CoA dehydrogenase [EC:1.1.1.35]	**Figure 1D**
K10797	enr; 2-enoate reductase [EC:1.3.1.31]	**Figure 1D**
K18244	mmgC; acyl-CoA dehydrogenase [EC:1.3.99.-]	**Figure 1D**

Asterisks indicate KOs with statistically significant differences between diet types.

### Variation in the mouse gut metagenome resulting from treatment associates with cognitive outcomes and ceramide concentrations in the brain and liver

We evaluated whether KO beta diversity was associated with the interaction between treatment and spatial learning and memory covariates in a linear regression model. Several spatial learning and memory variables explained mouse gut metagenome KO Bray-Curtis dissimilarity, alongside treatment (**Table 2**). L_Hid2 through L_Hid6 and P_TargetQ metrics showed differences related to gut microbiome diversity and treatment such that all supplement-associated microbiomes differed from the control. Gut microbiomes differed in association with P_cross and SS_Vis metrics such that DXN and TXN microbiomes had similar metrics to each other but were different from XN, and all differed from controls. P_time metrics were also associated with gut microbiome differences between XN and TXN, which were distinct from controls (whereas DXN was not). Overall, we find that these measures of mouse ability to locate a hidden platform were associated with microbiome functional capacity changes due to supplement regimes.

72 KOs changed significantly in abundance with task (visible platform) and spatial (hidden platform) learning covariates in a treatment-dependent manner (random forest regression, p ≤ 0.05) (**Figure 2**; **Supplementary Figure S1**). For example, K21600 (CsoR family transcriptional regulator, copper-sensing transcriptional repressor) had a positive association with L_Vis1 scores in control and XN-supplemented mice, although XN-supplemented mice had a lower average (**Figure 2B**). K21600 abundance also exhibited a negative association with L_Vis1 scores in DXN- and TXN-supplemented mice, with lower L_Vis1 scores in TXN-supplemented mice (**Figure 2B**). K00566 (tRNA-uridine 2-sulfurtransferase) had a negative association with L_Hid6 scores in control samples, but positive associations with L_Hid6 scores, which were lower than control scores, for all three supplemented samples (**Figure 2B**). K01817 (tryptophan biosynthesis, chorismate to tryptophan), is related to tryptophan, a known signaling molecule between the gut and brain. This gene’s abundance increased with L_Hid6 score, and the scores were lower than the control group, for mice whose diet was supplemented with XN and DXN, with XN-supplemented mice exhibiting the steepest slope between L_Hid6 score and K01817 abundance.

**Figure 5:**
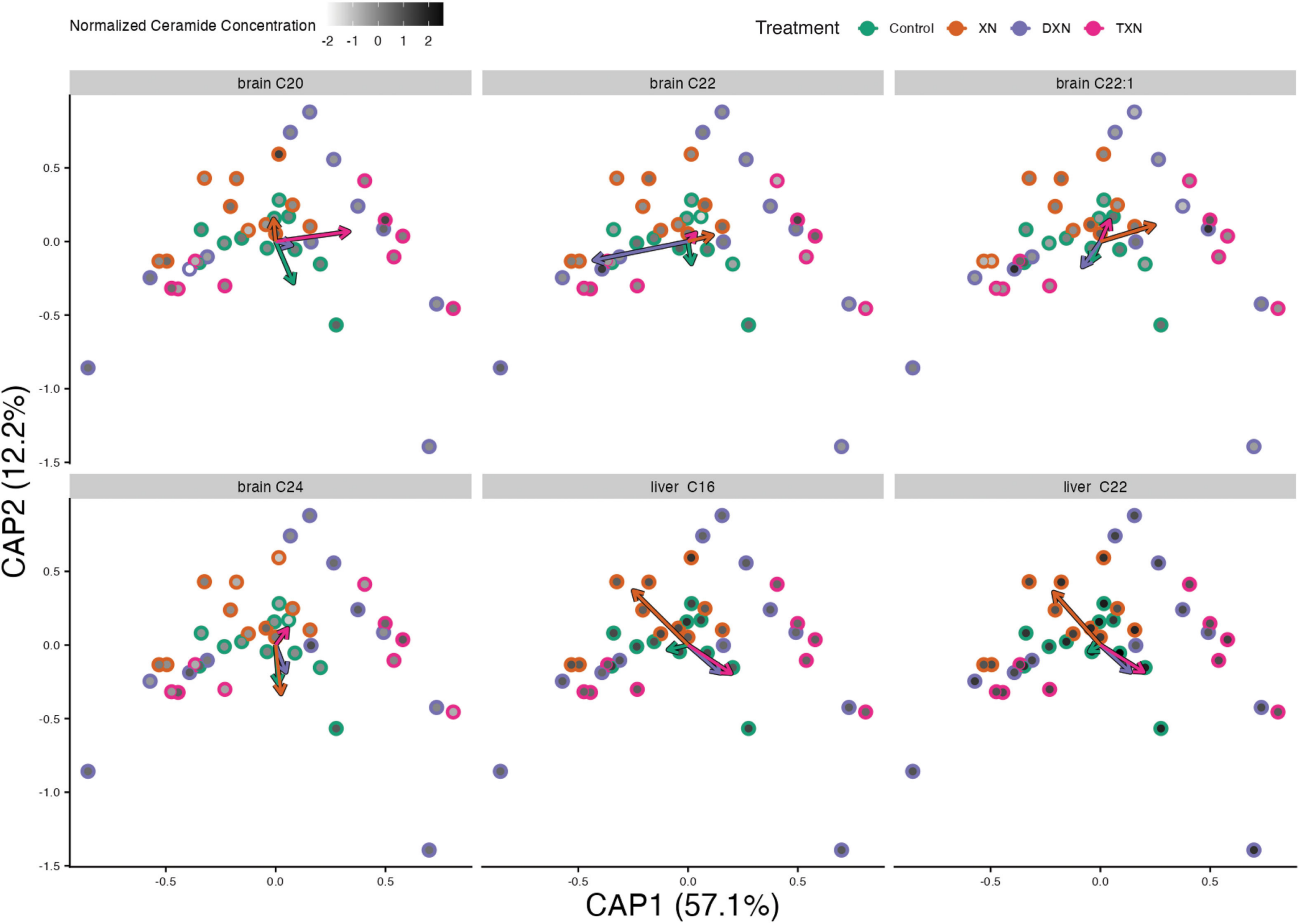
Gut microbiome beta diversity correlates with treatments and host ceramides. dbRDA ordinations illustrate that brain and liver ceramide concentrations explain Bray-Curtis dissimilarity of KOs in mouse gut metagenomes in a treatment-dependent manner. Points are shared across all panels (each is one mouse gut metagenome in a fixed ordination space); only the fitted ceramide vectors differ between panels. Arrows indicate fitted vectors, which indicate the direction and magnitude of each ceramide’s association with community beta-diversity (indicated by panel label) by treatment interaction term from the PERMANOVA results. Both circles and arrows are colored by treatment. Percent variance in Bray-Curtis dissimilarity explained is presented in parentheses in the axis labels.

Inhibition of ceramide synthesis prevents weight gain, glucose intolerance in diabetes models, and obesity-mediated cognitive impairment (Holland et al., 2007; Paraiso et al., 2021; Turpin et al., 2014). Therefore, we evaluated whether KO beta-diversity was associated with the interaction between treatment and brain and/or liver ceramides. Our analysis found that four brain and two liver ceramides associated with beta-diversity in a manner that depended on treatment (**Table 3**; **Figure 5**). Brain C20 and C22 ceramides differed in association with gut microbiome functional beta diversity between all supplement regimes and against the control. Brain C22:1 differed in association with gut microbiome beta diversity between XN and TXN, which also were distinct from controls. Brain C24 only changed with gut microbiome beta diversity in TXN-supplemented mice. Lastly, liver C16 and C22 ceramides were both altered alongside gut microbiome beta diversity in all supplemented mice compared to controls, with XN differing from DXN and TXN (the non-estrogenic derivatives being similar). While our hypothesis concerned ceramides broadly rather than any specific species, C22 emerged from this analysis as the only ceramide whose association with XN-related gut microbiome differences overlapped in both liver and brain. We therefore highlight it as a *post hoc* candidate biomarker. This analysis indicates that variation in the gut microbiome associates with variation in the host lipidome, especially with respect to brain and liver ceramides, and that XN metabolites may modulate this relationship. We then used random forest to identify KOs predicting ceramide levels across different treatments. The model identified 87 significant KOs (**Supplementary Table 3**). As a standout example, we present linear regression results in two KOs predicting C22 ceramide levels in the liver and brain (**Figure 3**). K03338, which had lower abundance in the liver with TXN and DXN, is a 5-dehydro-2-deoxygluconokinase, which participates in inositol metabolism (Anderson and Magasanik, 1971). K06394 is a sporulation protein (Serrano et al., 2008), which had lower abundance in the brain with DXN supplementation.

Our previous research suggested ceramide levels were linked to secondary bile acid synthesis (Paraiso et al., 2021; Zhang et al., 2020), which could explain cognitive shifts. Gaussian linear modeling identified relationships between two microbial (secondary) bile acids (HDCA and DCA-isomer) and TXN supplementation, but no behavioral associations were significant in interaction with the treatment. TXN supplementation was associated with lower abundance of both HDCA and DCA-isomer (coefficient = -3.2, p = 0.049 and coefficient = -3.2, p = 0.009, respectively) (**Figure 4**). This observation extends our prior TXN-bile acid findings (Zhang et al., 2020), though bile acid levels did not independently associate with behavior here.

## Discussion

**Figure 6:**
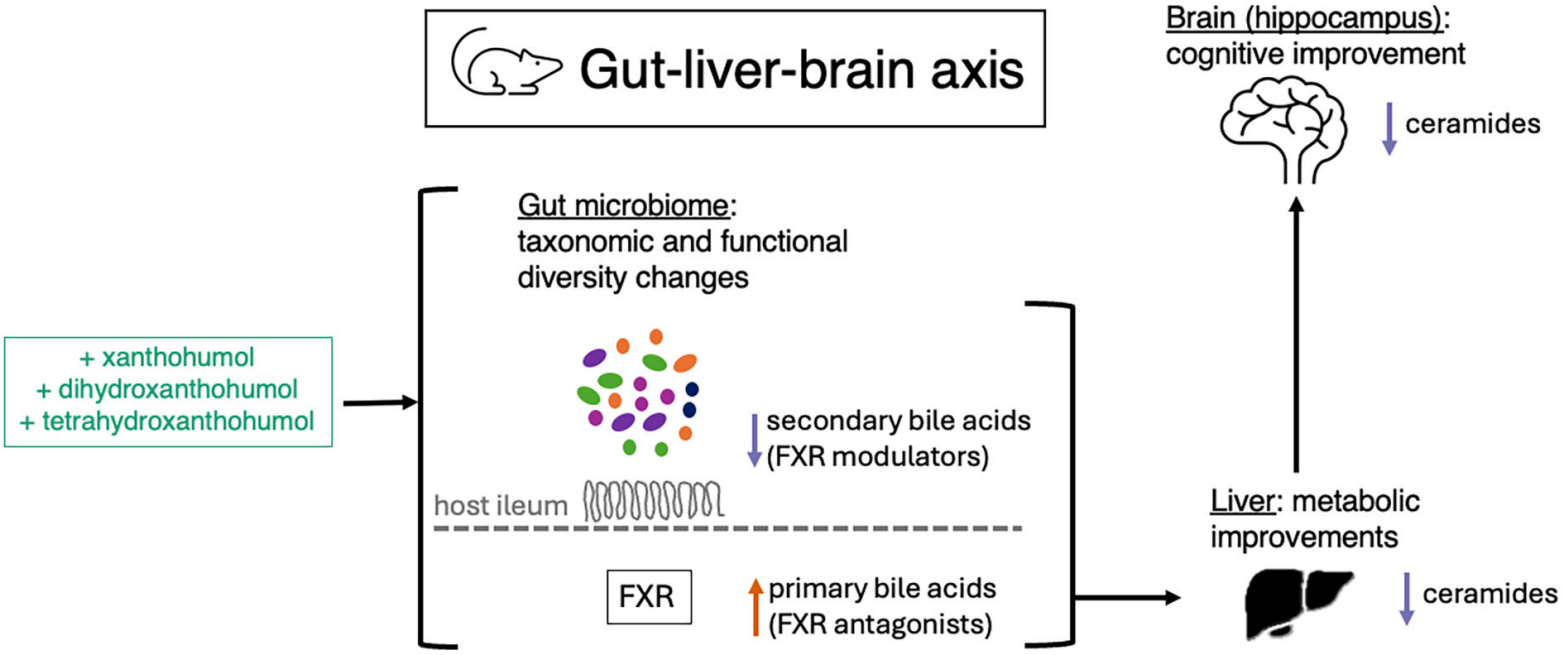
A conceptual model of our present results alongside findings derived from our previous body of work (Jamieson et al., 2025; Kundu et al., 2022, 2022; Miranda et al., 2018; Paraiso et al., 2025, 2021, 2020, 2019; Zhang et al., 2020). Past work suggested XN and its derivatives regulate the gut-liver-brain axis via bile acid and ceramide metabolism. We identified gut microbial genes associated with cognitive and ceramide profiles in relation to this model, and additionally observed treatment-associated differences in microbially-derived genes that warrant further investigation.

Our findings are consistent with the hypothesis that dietary supplementation with XN or its non-estrogenic derivatives, DXN and TXN, influences cognitive outcomes through interactions involving the gut microbiome and host ceramide metabolism. By integrating shotgun metagenomic data with prior evidence of cognitive improvement in the same experimental animals (Miranda et al., 2018), we identified microbial gene associations potentially underlying supplement benefits. Each supplement was linked to distinct changes in gut microbiome functional capacity, with DXN and TXN producing more pronounced effects than XN. This was consistent with previous reports that hydrogenated derivatives more effectively reduce adipose inflammation and alter bile acid metabolism in association with compositional changes to the gut microbiome (Newman et al., 2023; Zhang et al., 2020). TXN supplementation also reduced levels of two microbially produced bile acids. These microbiome shifts align with our understanding of previously reported improvements in spatial learning and memory retention (Kundu et al., 2022; Miranda et al., 2018; Paraiso et al., 2020), and support a model in which XN-induced microbial changes affect secondary bile acid production, which may inhibit host farnesoid X receptor (FXR) signaling, thus decreasing host ceramides production and improving cognitive function (Jamieson et al., 2025; Kundu et al., 2022, 2022; Miranda et al., 2018; Paraiso et al., 2025, 2021, 2020, 2019; Zhang et al., 2020) (**Figure 6**). The present shotgun metagenomic analysis extends this model to the level of microbial gene function. (**Figure 6**).

TXN-specific reduction in HDCA and DCA-isomer recapitulates our prior observations of XN-derivative effects on microbial bile acid metabolism (Zhang et al., 2020). Although these bile acids did not independently associate with behavior, their reduction under TXN, the compound most divergent from control, suggests XN metabolites reshape multiple microbial outputs that converge on host FXR signaling. Tryptophan-derived metabolites themselves modulate conjugated secondary bile acid pools and FXR activity (Chen et al., 2024), suggesting that the tryptophan and bile acid signals we observe may reflect linked rather than independent microbial responses to supplementation.

Our analysis also revealed significant associations between gut microbial gene profiles and ceramide concentrations in hepatic and/or neural tissues, suggesting that gut microbiome function is linked to ceramide metabolism in the liver and brain. One ceramide, C22, was associated as such in both the liver and brain profiles. For example, in the liver and brain, two KOs, one involved in inositol synthesis, and one involved in sporulation, were associated with C22 (**Figure 3**). The inositol synthesis KO is of particular interest, as inositol deficiency has been linked to insulin resistance and functioning in patients with diabetes mellitus (Ostlund et al., 1993) and polycystic ovarian syndrome (Bevilacqua and Bizzarri, 2018). Inositol is also linked with neurotransmitter modulation in panic, depression, and obsessive compulsive disorder (Harvey et al., 2002), though whether this connection is relevant to the gut microbiome-ceramide associations observed here remains speculative. The abundance of this KO was lower in DXN and TXN fed mice compared to control and was negatively associated with C22 ceramide levels in DXN mice but positively associated in TXN mice. Notably, modulation of ceramide synthesis pathways via dietary interventions mitigates cognitive decline linked to high-fat diet and/or metabolic dysfunction (Kundu et al., 2022; Paraiso et al., 2020). Together these findings are consistent with a link between gut microbiome-derived inositol synthesis (and other KOs), ceramide metabolism, and insulin function; any extension to cognition remains hypothesis-generating and would require targeted mechanistic studies in the context of XN supplementation.

One way in which ceramides may link gut microbiota and cognition is via host signaling pathways that integrate multiple microbial inputs, most notably FXR and nuclear factor kappa-light-chain-enhancer of activated B cells (NF-κB). FXR regulates hepatic and intestinal lipid and bile acid metabolism pathways (Chen et al., 2024; Fleishman and Kumar, 2024; Jiang et al., 2015, p. 20; Li et al., 2023; Paraiso et al., 2025, 2021; She et al., 2022, p. 202; Xie et al., 2017), and is implicated in mediating the cognitive effects of XN in HFD-fed mice (Kundu et al., 2022; Miranda et al., 2018; Paraiso et al., 2020). Studies with FXR knockout mice demonstrated that receptors in the intestine and the liver affect ceramide synthesis and XN-associated cognitive improvements (Kundu et al., 2022). FXR also responds directly to gut microbial metabolites, including microbial secondary bile acids (Paraiso et al., 2025), including those derived from tryptophan metabolism (Chen et al., 2024; She et al., 2022). In parallel, NF-κB signaling plays a central role in inflammatory responses and gut barrier integrity, and XN supplementation modulates NF-κB activation, reducing inflammation and improving metabolic outcomes (Bradley et al., 2020). In our analysis, we identified several microbial genes related to oxidative stress that associated with supplementation and cognition, including a beta-oxidation gene that is involved in fatty acid catabolism. Although the specific fatty acids substrates of this gene are unknown, prior work shows that polyunsaturated fatty acids can act as ligands for FXR (Zhao et al., 2004), and excess saturated fatty acids are associated with ceramide synthesis and glucose intolerance (Holland et al., 2007; Summers, 2006). It is plausible that the XN-associated changes in gut microbiome metabolites exert beneficial effects on cognition and lipid homeostasis by engaging these host signaling pathways via the gut-liver-brain axis. Further delineating these interactions through targeted mechanistic experiments could yield novel therapeutic insights and deepen our understanding of microbiome-host crosstalk.

Although our findings offer novel insights, given inherent differences between murine and human physiology, their translational relevance requires further investigation. Further, the present work is correlational in nature, used to inform future experimental designs. Establishing a causal relationship between microbial changes, ceramide levels, and cognitive outcomes from supplementation will require targeted mechanistic studies. This includes microbiota transplantation experiments in supplemented germ-free animals, testing additional gut microbiota capable of metabolizing XN substrates (as in Paraiso et al., 2019), or integrated transcriptomic and metabolomic analyses to determine the extent the microbiome directly contributes to bile acid and/or tryptophan metabolism. Because we did not quantify tryptophan, serotonin, or indole metabolites, nor assay the activity of the identified genes, any tryptophan-serotonin interpretation of these gene-level associations remains a hypothesis requiring direct metabolomic and functional validation.

In summary, this integrative, multi-omic analysis provides evidence that supplementation with XN and its hydrogenated derivatives modulates gut microbial functions in ways that are linked to host lipid metabolism and cognitive performance. These findings underscore the importance of considering microbiome functional capacity when developing dietary supplementation strategies to improve metabolic and cognitive health. Continued investigation of these microbiome-associated mechanisms and their interaction with host metabolism and neurotransmitter chemical cycles holds substantial promise for informing targeted nutritional therapies for cognitive impairment associated with metabolic disorders.

## Data Availability

The datasets presented in this study can be found in online repositories. The names of the repository/repositories and accession number(s) can be found in the article/**Supplementary Material**.

## Supplementary material

Supplementary Figure 1 Heatmap of KOs whose abundances are significantly associated with behavior. We took the list of KOs identified as “important” in a random forest regression model in interaction with XN supplement type and ran optimized regressions (Gaussian, polynomial, binomial) identifying which KOs were significantly associated with behavior metrics (behavior ~ KO abundance * treatment). Each square’s color represents whether a significant association was identified between KO abundance and the behavior metric with XN metabolite. KOs are shown on the y-axis and behavior metrics are shown on the x-axis. As a reminder, L is latency, P is platform, SS is for swim speed, representing the different types of behavior tests run (see methods for expanded explanation of behavior metrics).

Supplementary Table 1 Model fit characteristics for best fit random forest regression model identifying KOs predicting each behavior characteristic and ceramide (either from hippocampus or liver tissue samples). KO data was filtered on prevalence thresholds 0, 20, 40, 60, 80, and 95%, and a random forest model was run for each of these data sets. The best fit model (based on RMSE, R^2^, and MSE) of these models is reported in the main manuscript.

Supplementary Table 2 PERMANOVA results for beta-diversity metrics by diet treatment for both taxonomic annotations (assigned via Kraken2) of mouse gut metagenomes.

Supplementary Table 3 KOs predicting ceramide levels via random forest regression model (p-values ≤ 0.05). Information is listed about each KO name and existing descriptive information in the KEGG database, as well as what ceramide and tissue type they were associated with.
