# Supplementary material for: Xanthohumol and its non-estrogenic derivatives link to the gut-liver-brain axis to improve cognition in mice with diet-induced obesity
Source: Front Physiol. 2026 Aug 5;17:1886058. doi: 10.3389/fphys.2026.1886058 (PMC13485733; doi:10.3389/fphys.2026.1886058)
Supplement: Supplementary file 1 [file SupplementaryFile1.docx]

**Supplemental figures and tables**

Supplemental Table 1: Model fit characteristics for best fit random forest regression model identifying KOs predicting each behavior characteristic and ceramide (either from hippocampus or liver tissue samples). KO data was filtered on prevalence thresholds 0, 20, 40, 60, 80, and 95%, and a random forest model was run for each of these data sets. The best fit model (based on RMSE, R^2^, and MSE) of these models is reported in the main manuscript.

| Covariates | Prevalence filter | RMSE | R^2^ | MSE |
| --- | --- | --- | --- | --- |
| L_Vis1 | 0 | 13.22529 | 0.13966332 | 11.10714 |
| L_Vis2 | 95 | 7.573207 | 0.09034877 | 6.065677 |
| L_Hid1 | 80 | 9.843777 | 0.16219907 | 6.882255 |
| L_Hid2 | 0 | 17.29197 | 0.06896751 | 15.34811 |
| L_Hid3 | 60 | 14.74159 | 0.08466239 | 13.18742 |
| L_Hid4 | 95 | 17.12354 | 0.306547 | 15.83417 |
| L_Hid5 | 60 | 19.16315 | 0.1866512 | 17.00117 |
| L_Hid6 | 95 | 15.97247 | 0.1963542 | 13.30308 |
| L_Vis3 | 95 | 12.85941 | 0.1548481 | 9.751352 |
| L_Vis4 | 0 | 5.472701 | 0.11255631 | 4.075332 |
| P_Time | 60 | 15.92835 | 0.1747139 | 13.13443 |
| P_Cross | 95 | 1.251405 | 0.05899789 | 0.9888759 |
| P_TargetQ | 40 | 0.0831112 | 0.1252222 | 0.06973584 |
| SS_Vis | 80 | 2.508955 | 0.06892424 | 1.876588 |
| C16 ceramide in brain | 0 | 1.17737756 | 0.13180577 | 0.87688263 |
| C18 ceramide in brain | 0 | 0.86230648 | 0.2215237 | 0.67616582 |
| C20 ceramide in brain | 95 | 0.54083265 | 0.11287024 | 0.38675735 |
| C22:1 ceramide in brain | 95 | 0.58711719 | 0.07845262 | 0.42962789 |
| C22 ceramide in brain | 0 | 0.5246528 | 0.12869041 | 0.4171558 |
| C24:1 ceramide in brain | 80 | 0.53285975 | 0.25909416 | 0.43561475 |
| C24 ceramide in brain | 20 | 0.56735965 | 0.12438266 | 0.44698826 |
| C16 ceramide in liver | 20 | 0.23118004 | 0.51847269 | 0.17272518 |
| C18 ceramide in liver | 0 | 0.32626087 | 0.37122766 | 0.26048527 |
| C20 ceramide in liver | 0 | 0.34202873 | 0.34930039 | 0.26147337 |
| C22 ceramide in liver | 95 | 0.29288433 | 0.58791433 | 0.24607675 |
| C24 ceramide in liver | 95 | 0.07862728 | 0.41475645 | 0.0645147 |

Supplemental Table 2: PERMANOVA results for beta-diversity metrics by diet treatment for both taxonomic annotations (assigned via Kraken2) of mouse gut metagenomes.

| Beta-diversity metric | Term | df | Sum of squares | R2 | statistic | p value |
| --- | --- | --- | --- | --- | --- | --- |
| Bray-Curtis | Diet | 3 | 0.97 | 0.16 | 2.80 | 0.014 |
|  | Residual | 43 | 4.95 | 0.84 |  |  |
|  | Total | 46 | 5.92 | 1.00 |  |  |
| Canberra | Diet | 3 | 0.80 | 0.25 | 4.77 | 0.001 |
|  | Residual | 43 | 2.40 | 0.75 |  |  |
|  | Total | 46 | 3.20 | 1.00 |  |  |
| Sørensen | Diet | 3 | 0.00 | 0.22 | 3.98 | 0.001 |
|  | Residual | 43 | 0.01 | 0.78 |  |  |
|  | Total | 46 | 0.02 | 1.00 |  |  |
|  |  |  |  |  |  |  |

Supplemental Table 3: KOs predicting ceramide levels via random forest regression model (p-values ≤ 0.05). Information is listed about each KO name and existing descriptive information in the KEGG database, as well as what ceramide and tissue type they were associated with.

| KO number | p-value | Ceramide | Tissue sample | KO name | Description |
| --- | --- | --- | --- | --- | --- |
| K00003 | 1.87E-27 | C18 | liver | hom; homoserine dehydrogenase [EC:1.1.1.3] | Methionine biosynthesis, aspartate => homoserine => methionine; Threonine biosynthesis, aspartate => homoserine => threonine |
| K00244 | 1.92E-190 | C24 | liver | frdA; succinate dehydrogenase flavoprotein subunit [EC:1.3.5.1] | Citrate cycle (TCA cycle, Krebs cycle); Citrate cycle, second carbon oxidation, 2-oxoglutarate => oxaloacetate; Fumarate reductase, prokaryotes; Reductive citrate cycle (Arnon-Buchanan cycle) |
| K00433 | 9.80E-08 | C22 | brain | cpo; non-heme chloroperoxidase [EC:1.11.1.10] | NA |
| K00441 | 1.44E-128 | C16 | liver | frhB; coenzyme F420 hydrogenase subunit beta [EC:1.12.98.1] | NA |
| K00663 | 6.51E-46 | C16 | liver | aacA; aminoglycoside 6'-N-acetyltransferase [EC:2.3.1.82] | NA |
| K00759 | 9.63E-227 | C24 | liver | APRT, apt; adenine phosphoribosyltransferase [EC:2.4.2.7] | NA |
| K00805 | 3.55E-21 | C24 | liver | hepS; heptaprenyl diphosphate synthase component 1 [EC:2.5.1.30] | NA |
| K00868 | 1.17E-15 | C24 | brain | pdxK, pdxY; pyridoxine kinase [EC:2.7.1.35] | NA |
| K00945 | 2.93E-42 | C18 | brain | cmk; CMP/dCMP kinase [EC:2.7.4.25] | NA |
| K01034 | 4.21E-24 | C24 | brain | atoD; acetate CoA/acetoacetate CoA-transferase alpha subunit [EC:2.8.3.8 2.8.3.9] | NA |
| K01071 | 5.99E-35 | C16 | liver | MCH; medium-chain acyl-[acyl-carrier-protein] hydrolase [EC:3.1.2.21] | NA |
| K01232 | 3.80E-05 | C16 | liver | glvA; maltose-6'-phosphate glucosidase [EC:3.2.1.122] | NA |
| K01624 | 0.00025736 | C22 | brain | FBA, fbaA; fructose-bisphosphate aldolase, class II [EC:4.1.2.13] | Glycolysis (Embden-Meyerhof pathway), glucose => pyruvate; Gluconeogenesis, oxaloacetate => fructose-6P; Reductive pentose phosphate cycle (Calvin cycle); Reductive pentose phosphate cycle, glyceraldehyde-3P => ribulose-5P; Formaldehyde assimilation, xylulose monophosphate pathway |
| K01627 | 0.00954555 | C18 | brain | kdsA; 2-dehydro-3-deoxyphosphooctonate aldolase (KDO 8-P synthase) [EC:2.5.1.55] | CMP-KDO biosynthesis |
| K01633 | 2.35E-234 | C18 | liver | folB; 7,8-dihydroneopterin aldolase/epimerase/oxygenase [EC:4.1.2.25 5.1.99.8 1.13.11.81] | Tetrahydrofolate biosynthesis, GTP => THF; Tetrahydrofolate biosynthesis, mediated by ribA and trpF, GTP => THF |
| K01849 | 7.21E-17 | C24 | brain | E5.4.99.2B, mcmA2; methylmalonyl-CoA mutase, C-terminal domain [EC:5.4.99.2] | Hydroxypropionate-hydroxybutylate cycle; 3-Hydroxypropionate bi-cycle; Propanoyl-CoA metabolism, propanoyl-CoA => succinyl-CoA |
| K01928 | 0.0001874 | C20 | liver | murE; UDP-N-acetylmuramoyl-L-alanyl-D-glutamate--2,6-diaminopimelate ligase [EC:6.3.2.13] | NA |
| K02182 | 0.00254744 | C16 | brain | caiC; carnitine-CoA ligase [EC:6.2.1.48] | NA |
| K02358 | 0.00010911 | C24:1 | brain | tuf, TUFM; elongation factor Tu | NA |
| K02790 | 0.00690474 | C20 | liver | NA | NA |
| K03286 | 1.99E-06 | C16 | brain | TC.OOP; OmpA-OmpF porin, OOP family | NA |
| K03300 | 2.04E-48 | C16 | liver | TC.CITMHS; citrate-Mg2+:H+ or citrate-Ca2+:H+ symporter, CitMHS family | NA |
| K03335 | 0.0007337 | C18 | brain | iolE; inosose dehydratase [EC:4.2.1.44] | NA |
| K03336 | 7.10E-42 | C16 | liver | iolD; 3D-(3,5/4)-trihydroxycyclohexane-1,2-dione acylhydrolase (decyclizing) [EC:3.7.1.22] | NA |
| K03338 | 2.39E-06 | C22 | liver | iolC; 5-dehydro-2-deoxygluconokinase [EC:2.7.1.92] | NA |
| K03478 | 6.17E-10 | C22 | liver | chbG; chitin disaccharide deacetylase [EC:3.5.1.105] | NA |
| K03563 | 1.95E-07 | C16 | brain | csrA; carbon storage regulator | NA |
| K03574 | 3.62E-06 | C24 | liver | mutT, NUDT15, MTH2; 8-oxo-dGTP diphosphatase [EC:3.6.1.55] | NA |
| K03647 | 1.65E-215 | C18 | liver | nrdI; protein involved in ribonucleotide reduction | NA |
| K03693 | 6.51E-46 | C16 | liver | pbp1b; penicillin-binding protein 1B | NA |
| K03734 | 8.83E-37 | C18 | brain | apbE; FAD:protein FMN transferase [EC:2.7.1.180] | NA |
| K05520 | 0.01102743 | C24 | brain | pfpI; protease I [EC:3.5.1.124] | NA |
| K05795 | 2.20E-08 | C20 | liver | terD; tellurium resistance protein TerD | NA |
| K05799 | 3.37E-55 | C22:1 | brain | pdhR; GntR family transcriptional regulator, transcriptional repressor for pyruvate dehydrogenase complex | NA |
| K05919 | 0.00331627 | C22 | liver | dfx; superoxide reductase [EC:1.15.1.2] | NA |
| K06158 | 0.00057255 | C16 | brain | ABCF3; ATP-binding cassette, subfamily F, member 3 | NA |
| K06317 | 0.00724985 | C24 | liver | bofA; inhibitor of the pro-sigma K processing machinery | NA |
| K06714 | 2.40E-48 | C18 | brain | rocR; arginine utilization regulatory protein | NA |
| K06894 | 9.71E-32 | C24 | brain | yfhM; alpha-2-macroglobulin | NA |
| K06907 | 0.02407243 | C20 | liver | K06907; uncharacterized protein | NA |
| K06958 | 4.31E-14 | C22:1 | brain | rapZ; RNase adapter protein RapZ | NA |
| K07034 | 3.04E-186 | C24 | liver | K07034; uncharacterized protein | NA |
| K07120 | 1.64E-07 | C22 | liver | K07120; uncharacterized protein | NA |
| K07221 | 7.77E-07 | C22:1 | brain | oprO_P; phosphate-selective porin OprO and OprP | NA |
| K07308 | 0.00020872 | C18 | brain | dmsC; anaerobic dimethyl sulfoxide reductase subunit C | NA |
| K07309 | 4.43E-13 | C24 | brain | ynfE; Tat-targeted selenate reductase subunit YnfE [EC:1.97.1.9] | NA |
| K07685 | 3.08E-10 | C22:1 | brain | narP; two-component system, NarL family, nitrate/nitrite response regulator NarP | NA |
| K07714 | 0.03282623 | C24:1 | brain | atoC; two-component system, NtrC family, response regulator AtoC | NA |
| K07714 | 1.14E-220 | C16 | liver | atoC; two-component system, NtrC family, response regulator AtoC | NA |
| K07719 | 1.81E-126 | C16 | liver | glnL; two-component system, response regulator GlnL | NA |
| K08298 | 0.0117374 | C24:1 | brain | caiB; L-carnitine CoA-transferase [EC:2.8.3.21] | NA |
| K08676 | 2.20E-08 | C20 | liver | tri; tricorn protease [EC:3.4.21.-] | NA |
| K09131 | 7.00E-08 | C18 | liver | K09131; uncharacterized protein | NA |
| K09815 | 0.00227382 | C24 | liver | znuA; zinc transport system substrate-binding protein | NA |
| K09835 | 9.39E-167 | C22:1 | brain | crtISO, crtH; prolycopene isomerase [EC:5.2.1.13] | beta-Carotene biosynthesis, GGAP => beta-carotene |
| K10212 | 1.40E-31 | C16 | brain | K10212, crtO; glycosyl-4,4'-diaponeurosporenoate acyltransferase [EC:2.3.1.-] | NA |
| K10536 | 1.92E-05 | C22:1 | brain | aguA; agmatine deiminase [EC:3.5.3.12] | NA |
| K10547 | 0.0181418 | C20 | liver | gguB; putative multiple sugar transport system permease protein | NA |
| K11183 | 0.01569535 | C22 | brain | fruB, fpr; multiphosphoryl transfer protein [EC:2.7.1.202] | NA |
| K12267 | 2.10E-20 | C22:1 | brain | msrAB; peptide methionine sulfoxide reductase msrA/msrB [EC:1.8.4.11 1.8.4.12] | NA |
| K13290 | 3.45E-136 | C22:1 | brain | K13290; serine---pyruvate transaminase [EC:2.6.1.51] | NA |
| K13963 | 2.00E-61 | C18 | brain | SERPINB; serpin B | NA |
| K14645 | 1.01E-05 | C22 | liver | K14645; serine protease [EC:3.4.21.-] | NA |
| K15383 | 4.18E-11 | C24 | brain | K15383; MtN3 and saliva related transmembrane protein | NA |
| K15739 | 4.83E-24 | C18 | brain | vanB, vanA, vanD; D-alanine---(R)-lactate ligase [EC:6.1.2.1] | Vancomycin resistance, D-Ala-D-Lac type |
| K16950 | 5.63E-111 | C24 | liver | asrA; anaerobic sulfite reductase subunit A | NA |
| K17204 | 0.01820864 | C20 | brain | eryE; erythritol transport system ATP-binding protein | NA |
| K17242 | 0.00170461 | C20 | liver | aguF; alpha-1,4-digalacturonate transport system permease protein | NA |
| K17733 | 4.78E-37 | C22:1 | brain | cwlK; peptidoglycan LD-endopeptidase CwlK [EC:3.4.-.-] | NA |
| K17899 | 7.17E-09 | C20 | liver | oraS; D-ornithine 4,5-aminomutase subunit alpha [EC:5.4.3.5] | NA |
| K18095 | 2.01E-32 | C22:1 | brain | mexY, amrB; multidrug efflux pump | Multidrug resistance, efflux pump MexXY-OprM |
| K18475 | 2.05E-57 | C24 | brain | fliB; lysine-N-methylase [EC:2.1.1.-] | NA |
| K18930 | 0.03048844 | C20 | liver | dld; D-lactate dehydrogenase | NA |
| K19302 | 3.78E-73 | C18 | brain | bcrC; undecaprenyl-diphosphatase [EC:3.6.1.27] | NA |
| K20268 | 5.44E-06 | C22 | brain | rhiA; rhizosphere induced protein | NA |
| K20268 | 2.03E-18 | C24 | brain | rhiA; rhizosphere induced protein | NA |
| K20989 | 0.02929877 | C20 | liver | DUR3; urea-proton symporter | NA |
| K21401 | 0.00076578 | C24 | brain | menJ; menaquinone-9 beta-reductase [EC:1.3.99.38] | NA |
| K22044 | 1.98E-178 | C16 | liver | ybiO; moderate conductance mechanosensitive channel | NA |
| K22224 | 1.62E-99 | C16 | brain | acdB; acetate---CoA ligase (ADP-forming) subunit beta [EC:6.2.1.13] | NA |
| K22224 | 3.67E-38 | C18 | brain | acdB; acetate---CoA ligase (ADP-forming) subunit beta [EC:6.2.1.13] | NA |
| K22299 | 0.00014751 | C16 | brain | rghR; HTH-type transcriptional regulator, competence development regulator | NA |

Supplemental Figure 1: Heatmap of KOs whose abundances are significantly associated with behavior. We took the list of KOs identified as “important” in a random forest regression model in interaction with XN supplement type and ran optimized regressions (Gaussian, polynomial, binomial) identifying which KOs were significantly associated with behavior metrics (behavior ~ KO abundance * treatment). Each square’s color represents whether a significant association was identified between KO abundance and the behavior metric with XN metabolite. KOs are shown on the y-axis and behavior metrics are shown on the x-axis. As a reminder, L is latency, P is platform, SS is for swim speed, representing the different types of behavior tests run (see methods for expanded explanation of behavior metrics).
